# The new age of play audiometry: prospective validation testing of an iPad-based play audiometer

**DOI:** 10.1186/1916-0216-42-21

**Published:** 2013-03-11

**Authors:** Jeffrey Yeung, Hedyeh Javidnia, Sophie Heley, Yves Beauregard, Sandra Champagne, Matthew Bromwich

**Affiliations:** 1 Division of Otolaryngology-Head and Neck Surgery, Children’s Hospital of Eastern Ontario, University of Ottawa, Ottawa, ON, Canada; 2 Children’s Hospital of Eastern Ontario, 401 Smyth Road, Ottawa, ON, K1H 8L1, Canada

**Keywords:** Hearing loss, Play audiometry, Pediatric

## Abstract

**Objective:**

The timely diagnosis of hearing loss in the pediatric population has significant implications for a child’s development. However, audiological evaluation in this population poses unique challenges due to difficulties with patient cooperation. Though specialized adaptations exist (such as conditioned play audiometry), these methods can be time consuming and costly. The objective of this study was to validate an iPad-based play audiometer that addresses the shortcomings of existing audiometry.

**Methods:**

We designed a novel, interactive game for the Apple® iPad® that tests pure tone thresholds. In a prospective, randomized study, the efficacy of this tool was compared to standard play audiometry. 85 consecutive patients presenting to the Audiology Clinic at the Children’s Hospital of Eastern Ontario (ages 3 and older) were recruited into this study. Their hearing was evaluated using both tablet and traditional play audiometry.

**Outcome measure:**

Warble-tone thresholds obtained by both tablet and traditional audiometry.

**Results:**

The majority of children in this age group were capable of completing an audiologic assessment using the tablet computer. The data demonstrate no statistically significant difference between warble-tone thresholds obtained by tablet and traditional audiometry (p=0.29). Moreover, the tablet audiometer demonstrates strong sensitivity (93.3%), specificity (94.5%) and negative predictive value (98.1%).

**Conclusion:**

The tablet audiometer is a valid and sensitive instrument for screening and assessment of warble-tone thresholds in children.

## Background & rationale

The timely diagnosis of hearing loss in children has significant implications for a child’s social and cognitive development. In young children, hearing loss is often inconspicuous resulting in delayed diagnosis and rehabilitation. As a result, children are at increased risk of delayed speech acquisition and the subsequent long-term sequelae. Speech delay and hearing loss also represents a significant cost to society due to the resources required for their treatment, such as special education, speech therapy, and social services [1,2]. However, it is clear that early detection is the key to restoring normal speech development and a favorable long-term outcome [3,4].

Definitive diagnosis of hearing loss is typically made through audiologic assessment of pure-tone air, bone and speech thresholds. Traditionally, pure-tone thresholds are documented by asking the subject if they can hear tones of varying frequency and intensity. While auditory brainstem reflex and otoacoustic emissions testing remain the gold standard of hearing assessment, these investigations pose some difficulty in children. Currently, standard clinical audiometry is performed using non-portable hardware and access is therefore limited in developing countries, where hearing loss is more prevalent [5].

Automation of pure-tone audiometry offers several potential benefits [6]. Particularly, a portable, automated audiometer improves accessibility, creating the possibility of routine pure-tone audiometry in the primary care setting or remote communities [6-11]. Such a device may eventually permit a parent or teacher access to screening audiometry, resulting in earlier detection of hearing impairment. However, there may be significant differences between mean hearing thresholds determined though automated audiometry and manual audiometry [12-14]. As such, clinical validation of any novel automated audiometer is a necessity.

Pure-tone audiometry, regardless of automation or lack thereof, is a mundane task. It is especially challenging to perform in the pediatric population where short attention span and the extent of cognitive development can be limiting factors. Children with hearing impairment may find audiometric testing even more difficult. Several adaptations of pure-tone audiometry are used to evaluate hearing in children. These techniques include behavioral observation, visual re-enforcement, and conditioned play audiometry. While more successful than conventional pure-tone audiometry, these adaptations are resource intensive and typically require two specially trained audiologists to administer. Naturally, an ideal solution would capitalize on the advantages of automation, while maintaining clinical validity and age appropriateness.

With the success of the Apple® iPad® (Apple Inc., Cupertino, CA), it has become evident that touch interface computing enables even the youngest of users to interact intuitively with complex systems. It is not surprising then that portable tablet computers have been widely embraced in healthcare and education [15-19]. Thus, the tablet operating system appears to the optimal environment in which to develop portable, automated diagnostic applications targeted at children. Our group has developed the first such pediatric tablet-based play audiometer [20].

The purpose of this study is to (1) determine the accuracy of audiometric thresholds obtained by tablet audiometry compared to the accepted standard, conditioned play audiometry in children; and (2) describe our experiences with administering an iPad-based hearing assessment in the pediatric population.

We expect children ages 3 and above to be capable of completing a hearing assessment using the tablet audiometer. Further, we hypothesize that the tablet audiometer will be a sensitive screening test and that the audiometric thresholds obtained will closely correlate with conventional audiometric evaluation.

## Methods

### Tablet audiometer

Interactive audiometry is a novel paradigm in the assessment of hearing thresholds, in which the patient controls the presentation and pace of sound stimuli, rather than the audiologist. The tablet play audiometer is engineered as a decision-tree interactive game (yes/no paradigm) or as a two alternative forced choice interaction [20]. The two-alternative forced choice test paradigm is a psychophysical method for eliciting responses from a person about his or her experiences of a stimulus whereby the user is only given two possible responses. No option for “I don’t know” or “not sure” is given [21-25].

**Figure 1:**
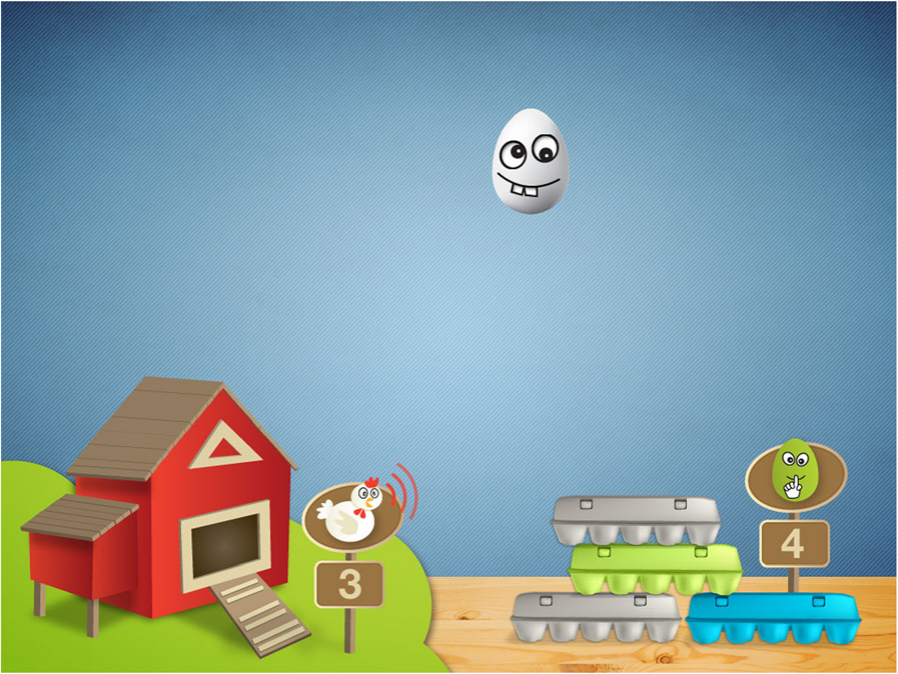
Tablet audiometer gameplay screenshot.

**Figure 2:**
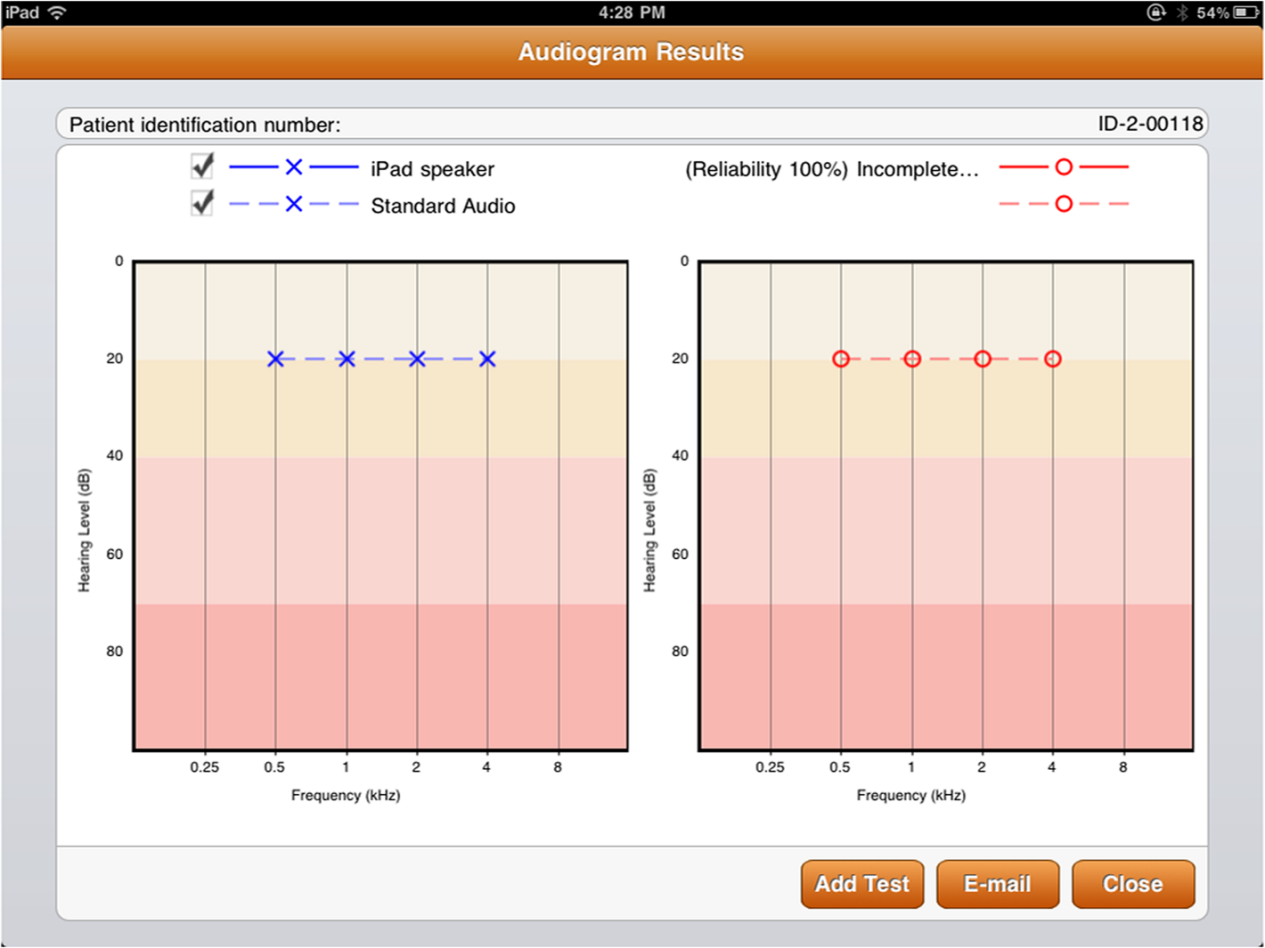
Tablet audiogram output.

In this test paradigm, the user is sequentially presented with a series of objects (eggs, for instance) and is tasked to categorize the objects into ‘sound-producing’ or ‘silent’ by dragging the objects into one of two ‘containers’ (i.e. chicken coop or egg carton) (Figure 1). In this “game”, the user is essentially navigating his/her own test and responding in a yes/no fashion to each stimulus. In another simplified version of the game, the child is only given one container and is tasked to drag the object into this container when it produces a sound. Each sound-producing object plays a unique warble-tone at either 500, 1000, 2000 or 4000 Hz. The intensity of sound decreases with each presentation until the child is unable to reproducibly sort the objects. Subsequently, the intensity of the sounds is increased. Thresholds are determined by the Hughson-Westlake method [26] in an up/down fashion to bracket the true threshold. Several “silent eggs” are also presented randomly as a measure of internal consistency (henceforth referred to as reliability). Once all frequencies are tested, a standard audiogram is obtained (Figure 2).

### Study population

Study participants were recruited from patients presenting to the Audiology Clinic at the Children’s Hospital of Eastern Ontario. The inclusion criteria for this study comprised children at least 3 years of age with normal or abnormal hearing. The exclusion criteria for the study included patients with previously diagnosed visual impairment, learning disability, or developmental delay. These patients were identified by a staff otolaryngologist (MB) through review of their patient records prior to enrollment. If the patient was eligible, informed consent was obtained from the parent/guardian. We aimed to enroll 80 patients into the study.

### Study design

A prospective, randomized study design was employed. Participants completed two sequential audiometric evaluations in a double walled sound booth, one with the tablet audiometer and one with traditional play audiometry assessing warble-tone thresholds. The order in which participants performed the two assessments was determined randomly by the flip of a coin. For the sake of simplicity and speed, unmasked air conduction was the sole investigation in this study. Sound field testing was performed if the child was not amenable to wearing headphones and binaural testing was performed in all other children. Stimuli were presented using TDH-39 headphones in both the tablet and audiologist test scenarios. When sound field testing was employed, the sound booth speaker system or the tablet speaker were used. An audiologist accompanied participants in both groups to provide motivation during the assessment.

This study was approved by the Children’s Hospital of Eastern Ontario Research Ethics Board #11/10x.

### Calibration

The tablet computers, headphones and speakers that were used in this study were professionally calibrated by Génie Audio Inc. (St-Laurent, QC) to ANSI S3.6-2004 standards [27].

### Sample size

Given the novel nature of both the method and the testing hardware no prior studies were available from which to calculate a sample size. A sample size of 80 consecutive patients was selected based on related publications comparing automated hearing tests to standard audiometry [7,10,11]. Five additional children were recruited to allow for possible subject dropout.

### Outcome measures

The primary outcome measure consisted of warble-tone thresholds obtained by both the tablet audiometer and by standard play audiometry. Normal hearing was defined as a threshold of less than or equal to 25 dB in each of the 4 test frequencies (500, 1000, 2000 or 4000 Hz). Hearing loss was defined as audiometric thresholds greater than 25 dB in any of 4 test frequencies. Secondary outcomes related to participant performance were also documented, including time to completion as well as feedback from the audiologists. In the tablet group, test reliability was evaluated by calculating the percentage of correctly sorted silent objects.

### Statistical analysis

Participants were excluded from analysis for (1) technical/gameplay issues (2) behavioural issues, or (3) questionable reliability, defined as incorrectly assigning greater than 50% of the silent objects. Descriptive statistics were used to summarize participant characteristics. Student’s t-test with Bonferroni correction was used to compare secondary outcomes between the two groups. Preliminary evaluation of the tablet’s performance was determined using a two-by-two table and calculations of sensitivity, specificity, positive and negative predictive value, and likelihood ratios positive were calculated.

A more intricate analysis of the concordance between the two hearing assessments was also performed. In order to test for overall differences between modalities (tablet versus play audiometry), a repeated measures model for the detection threshold in each ear at each frequency was fitted using linear mixed effects modeling. The model included fixed effects for testing modality (tablet versus play audiometry), frequency, and ear. In order to account for the correlation of thresholds within participants, random effects modeling was used for frequency nested within ear nested within participant.

## Results

85 consecutive patients who met the inclusion criteria were recruited into the study. 15 patients were excluded after hearing assessment, resulting in 70 subjects available for analysis. Fourteen of the 70 patients were tested using a sound field. The remainder (56) had binaural assessments for both hearing evaluations. The mean age of study participants was 5.2 years (range 3–13). The demographics for these individuals are summarized in Table 1.

**Table 1:** Participant demographics

	**Normal hearing**	**Abnormal hearing**
n	55	15
Mean age (years)	5.06 (range 3–9)	5.81 (range 3–13)
Duration of tablet assessment (secs)*	109±65	317±113
Number of objects*	23.8±9.45	67.1±19.1
Reliability (%)	90.4±22.4	92.0±10.8

± denotes standard deviation.

* denotes statistically significant difference.

Duration and number of objects for binaural assessment only.

Fifteen subjects were excluded from the analysis. Four were excluded from analysis due to poor reliability (<50%). Four patients were excluded for behavioural issues that prevented successful completion of the one or both hearing assessments. Seven patients were excluded for technical issues related to test administration. Of note, 10 of 11 subjects in the latter two categories had some degree of hearing loss. These results are summarized in Table 2.

**Table 2:** Excluded participants and rationale

**Study ID**	**Age (yrs)**	**Normal hearing?**	**Reason for exclusion**
**Poor reliability**			
2-007	4	Y	Reliability <50%
2-038	4	Y	Reliability <50%
2-043	4	Y	Reliability <50%
3-023	5	Y	Reliability <50%
**Technical/Gameplay issues**			
2-042	3	N	Incorrect speaker setting
2-060	7	N	Relied on visual cues
2-063	4	N	Poor headphone placement
2-066	4	N	Performed masked conditioned play audiometry
3-019	12	N	Performed masked conditioned play audiometry
2-050	3	N	Did not understand drag and drop
2-044	4	N	Did not understand drag and drop
**Behavioural issues**			
2-071	4	Y	Poor attention span
2-092	10	N	Poor attention span
2-108	4	N	Very slow. Poor attention span
2-011	3	N	Appeared to put all objects into one container

Overall, 55 patients were identified to have normal hearing by conditioned play audiometry, the traditionally accepted standard test. Of these, 52 were found to have normal hearing by tablet audiometry. The 3 remaining children scored slightly outside the defined parameters of normal hearing, namely a 30 dB threshold, in at least one frequency. This appeared to either be due to the child moving through the game too quickly or the presentation timing out before the child could make a decision.

The mean time to complete a binaural hearing test with the tablet was 152 s (SD±114 s) overall. In the group with normal hearing, the time was 109 s (SD±65 s). This increased to 317 s (SD±113 s) in children with abnormal hearing. The difference in test duration was statistically significant (p<0.0001).

53 patients in total had normal evaluation by tablet audiometry. One of these children was found to have a true mild hearing loss. This child appeared to understand the game but their results did not correlate well. The reliability score of this patient when using the table was only 75%. However he did not meet reliability criteria for exclusion (<50%). The results of the 2×2 table are summarized in Table 3.

**Table 3:** Comparison of tablet audiometer and conventional play audiometry

	**Play audiometry**	
	**Abnormal hearing**	**Normal hearing**
**Tablet audiometry**		
**Abnormal hearing**	14	3
**Normal hearing**	1	52

Sensitivity: 93.3% (95% CI = 71.7–99.6%).

Specificity: 94.5% (95% CI = 88.6–96.3%).

Positive predictive value: 82.3% (95% CI = 63.3–87.9%).

Negative predictive value: 98.1% (95% CI = 92.0–99.9%).

Positive likelihood ratio: 17.1 (95% CI = 6.31–26.7).

For the repeated measures analysis, 56 participants with valid results and binaural testing were available. Patients who were tested with a sound field were excluded from this particular analysis, as ear specific information was not available, making analysis impossible.

The model showed no significant effect of testing modality (compared to audiologists, the mean tablet threshold was 0.21 dB lower (95% CI=0.18 to 0.6 dB, p=0.29). The modeling was repeated for the abnormal cases (n=15), and for the normal cases (n=41). In both cases, the model showed no significant effect of testing modality. Compared to play audiometry, the mean tablet threshold was 1.13 dB lower (95% CI=0.27 to 2.52 dB, p=0.12) and 0.12 dB higher (95% CI=0.02 to 0.27 dB, p=0.10), respectively.

## Discussion

This paper describes the first trial of both a novel play algorithm using interactive audiometry and a new tablet audiometer. It is, to our knowledge, the first tablet-based, semi-automated, *play* audiometer to be used in a pediatric setting. The purpose of this study was two-fold, to validate the tablet audiometer as a child-friendly application for hearing assessment, as well as to compare tablet thresholds to the traditionally accepted standard play audiometry.

**Figure 3:**
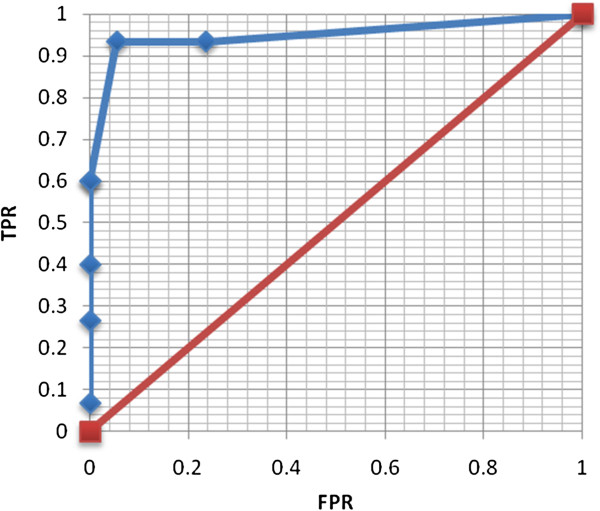
**Receiver operating characteristic curve for tablet audiometer.** Plot of the true positive rate (TPR) versus false positive rate (FPR) for varying definitions of normal hearing. The maximal TPR and minimal FPR are achieved at 25 dB. This demonstrates a high sensitivity and specificity for hearing loss.

The data reveal that the tablet audiometer produces warble-tone thresholds that are in agreement with the accepted standard (traditional play audiometry). This was achieved with narrow confidence intervals, suggesting sufficient statistical power. Audiometric data are acquired in an efficient manner, as demonstrated by a mean test duration of approximately 2.5 minutes. Moreover, the data reveals a high specificity 94.5% with a negative predictive value (NPV) of 98.1%, denoting that tablet audiometry is a robust screening tool. A positive likelihood ratio of 17.1 confirms the tablet audiometer’s capacity to diagnose hearing loss (Figure 3).

As the tablet audiometer is by definition an objective test, if calibrated adequately, it is not likely to be subject to issues with inter-rater reliability. However, it will be prudent in subsequent investigations to ensure strong inter-rater and test-retest reliability.

Conditioned play audiometry can often be employed with children as young as 2 years of age. The supervision and motivation given to the child by a second audiologist in the sound booth allows these very young children to be tested. In the current study, this second audiologist was present for both assessments (tablet and traditional) to maximize the successful completion of assessments, although attaining the appropriate level of support from the audiologists required training and experience.

Our data suggests that using the tablet interactive audiometry method, the majority (82%, 70/85) of children as young as 3 years of age are capable of understanding the concept of the game and completing the hearing assessment. In fact, 4 of the 15 patients were unable to complete the assessment due to a technical issue related to the audiologist. Therefore, 82% is a somewhat conservative assessment of the user-friendliness of the tablet audiometer. Despite the supervision of an audiologist however, some children had difficulties with the tablet audiometer. These difficulties resulted from technical deficiencies of the hardware/software (i.e. attempts to open other software, failure to understand ‘drag and drop’, becoming distracted by visual re-enforcements) and behaviors of the patient (i.e. boredom, poor comprehension of the game). A number of these difficulties eventually lead to the subject being excluded from the statistical analysis. Failure to complete the assessment also appeared to be more prevalent in children with abnormal hearing.

Several technical/gameplay issues were documented during data collection. In particular, children showed signs of fatigue with either test method quite quickly. During standard play audiometry, audiologists often switched games several times during standard play audiometry to keep the child engaged. By contrast, only two games were available when using the tablet, with the current software version. This stresses the importance of maintaining attention in this particular age group.

Furthermore, due to the nature of interactive audiometry, whereby the test is user-directed, action is required at each point in the decision tree. This gives the appearance of more decisions as compared to standard audiometry, where users who did not hear a sound were not required to perform an action. This was exacerbated in children with hearing loss, who were required to sort more objects in order to determine exact thresholds. For example, when testing a normal hearing individual, the minimum number of objects to complete an entire assessment was 16. This number increased to a maximum of 113 when hearing loss was present or unreliable results were being obtained. The average number of objects presented in in hearing loss was 67.1 (SD±19.1) (Table 1). For children with normal hearing, the average was 23.8 (SD±9.45) (p<0.0001). Despite these challenges, the vast majority of children were engaged enough to complete the tablet hearing assessment.

Some younger children were found to have difficulty understanding the concept of sorting. A simplified version of the game was also developed, where the child was only presented with one object and one container. The child placed the object in the container when it produced sound, and a timeout function advanced the game if the child did not. We found this allowed younger children to be accurately tested with the tablet audiometer.

Visual re-enforcements were originally included. Specifically, if the child sorted an object correctly, a pop-up star appeared. However, children with abnormal hearing tended to focus on these visual cues, ignoring the auditory cues (Table 2). Subsequent versions of the tablet game will optimize visual cues to maintain the user’s interest without distracting them.

The authors acknowledge several limitations to this study. Firstly, the majority of patients were normal hearing children. This is simply a reflection of the patient population when conducting sequential recruitment. However, a test population that is predominantly normal hearing will bias the study toward good correlation and successful completion of the relatively shorter hearing test. Second, the methods of our analysis excluded patients who were identified as having clear difficulty with the hearing assessment. This was done to ensure that the results reflect only the performance of the hardware. Although exclusion of ‘difficult’ patients limits the generalizability of our results, this analysis was deliberately used during this hardware validation phase. The proportion of patients excluded from analysis (18%, 15/85) insinuates a high degree of user-friendliness, especially given the potentially difficult patient population. Additionally, this emphasizes the importance of audiologist supervision, as the software is currently unable to determine if a child fully comprehends the game. Further gameplay refinement will likely increase the number of patients who are suitable candidates for tablet audiometry.

## Conclusion

The goal of our research is to develop a portable, versatile clinical audiometer designed for children. This study is the first to validate an automated play audiometer designed specifically for the pediatric population. In this study, we demonstrated that air conduction thresholds obtained by tablet audiometer are not significantly different from those obtained by standard play audiometry. The device has a strong predictive value for normal hearing and is highly sensitive for hearing loss. Finally, our results demonstrated that the tablet audiometer is a viable platform for testing children as young as 3 years of age. Future directions will focus on gameplay refinement and further validation testing of air vs. bone conduction thresholds and test-retest reliability.

## Competing interests

At the time of research the authors had no conflicts of interest to disclose, however the idea has since been patented.

## Authors’ contributions

JY was responsible for study design, statistical analysis and manuscript preparation. HJ was responsible for study design. SH, YB and SC were responsible for administration of audiometric testing and data collection/organization. MB is the senior author and involved in all levels of study design, data analysis and manuscript preparation. The manuscript was read and approved by all authors.

## Funding

CHEO AFP Innovation Fund

Department of Surgery Research Fund
